# Sulla (*Hedysarum coronarium* L.) Response to Drought Stress during Early Vegetative Stage

**DOI:** 10.3390/plants12193396

**Published:** 2023-09-26

**Authors:** Roberta Rossi, Mariana Amato, Salvatore Claps

**Affiliations:** 1 Council for Research in Agriculture and the Analysis of the Agricultural Economy, Research Centre for Animal Production and Aquaculture CREA-ZA, 85051 Potenza, Italy; salvatore.claps@crea.gov.it; 2 School of Agriculture, Forestry, Food and Environmental Sciences, University of Basilicata, 85100 Potenza, Italy; mariana.amato@unibas.it

**Keywords:** antihelmintic forage, *Hedysarum coronarium* L., drought stress, pot experiment

## Abstract

Sulla (*Hedysarum coronarium* L.) is a Mediterranean biannual anthelmintic forage. Due to its high productivity, nutraceutical value, and suitability for harsh environments, interest in this crop is growing. Under the current scenario of climate change and water scarcity, it is important to evaluate crop drought tolerance, especially for newly bred materials. Drought stress and well-watered conditions (50 vs. 80% of the field capacity) were applied in a pot experiment to compare responses of the widespread commercial variety Bellante with those of a recently released variety named ‘Centauro’, currently registered in the Italian national register of plant varieties but not yet available on the market. Compared to the well-watered treatment, drought-stressed plants showed lower values of fresh biomass (−69%), number of leaves (−68%), and root length (−49%). The Centauro cv. showed a different architecture to Bellante with more shoots (+43% *P* < 0.05) and a trend for more leaves (+25% *P* = 0.08). These traits are possibly related to its superior palatability. Centauro also developed a higher root length (+70%, *P* < 0.05) across irrigation levels. Drought stress affected condensed tannin (CT) content. A significant genotype × environment interaction was found with Centauro displaying more (+50%) and less (−35%) CT than Bellante under drought stress and well-watered conditions, respectively. The higher constitutive root length density of Centauro may be exploited in breeding programs aimed at improving the root sink, given the role of this trait in resource acquisition capacity and root-derived ecosystem services.

## 1. Introduction

Sulla (*Hedysarum coronarium* L.) is a biennial forage legume autochthonous of the Mediterranean basin and is a nutritious forage suitable for grazing, hay, and ensiling. In Europe, it is grown in an area spanning from Portugal to Greece. Suitable for harsh environments, it is a key forage in northern Africa (Morocco, Algeria, Tunisia), Egypt, Turkey, and Lebanon [1]. Research in Tunisia has shown how reseeding sulla into degraded semi-arid rangelands improves forage yield, land pastoral value, and water productivity [2] and considerably reduces overland flow and soil erosion compared to fallow or wheat [3].

In Italy until the late 1990s, sulla was second in importance to alfalfa for forage production; the cultivated area extended over 300,000 ha, most of which was located in southern regions [4]. During the 1980s, this crop was introduced in Australia and New Zealand where it rapidly gained popularity not only for forage production but also for land amelioration [5,6]. Sulla is a relatively high-yielding forage (with peak productions during the second year of establishment) up to the point of being considered a dual-purpose crop for both protein and biofuel production [7]. Borreani et al. [8] quantified forage yield and quality at different morphological stages during different years and at different locations. The dry matter yield ranged from 2 to more than 10 t ha^−1^ from the early vegetative stage to seed set. The protein content varied between 10 and 29%, being higher during early vegetative stages. Organic matter digestibility is highly variable (between 398 and 846 g kg^−1^), it decreases with plant age and size, and it is influenced by soil water content. Due to a moderate content of condensed tannin (CT), sulla is also an effective anti-bloating, anthelmintic forage [9,10,11,12]. This may open new markets for this crop especially in the organic sector due to the global concern over the presence of medicinal residues in animal products and the increasing prevalence of anthelmintic-resistant nematode strains. The presence of CT also affects animal product quality. Condensed tannins are powerful antioxidants capable of shielding dietary proteins and omega-3 fatty acids (FAs) from rumen degradation. Feeding ewes and goats on sulla improved both milk and meat FA profiles [13,14]. Sulla contains several health-promoting compounds and secondary metabolites: the alpha-linolenic acid content ranges between 462 and 419 g kg^−1^ total FA in fresh forage and silage, respectively, with values around 368 g kg^−1^ total FA in hay; leaves and flowers contain carotenoids and alpha-tocopherol (between 60 and 70 mg kg^−1^ DM) in fresh forage and silage, with 13 mg kg^−1^ DM in hay [15]. A detailed chemical analysis of wild sulla specimens collected at various locations in the south of Italy allowed the identification of a wide range of secondary metabolites belonging to the classes of flavonoids, proanthocyanidins, and saponins supporting the use of leaves and flowers as valuable sources of health-promoting compounds [16]. Sulla is also considered a land ameliorative crop, not only for its soil nitrogen fixation potential [17] but also for its effects on soil biophysical quality. Erosion studies in northern Tunisia showed that cultivating sulla on slopes between 4 and 12% increased water infiltration rates and reduced runoff by about 6 and 7 times compared to fallow and wheat, respectively [3]. Green manure from sulla also showed an interesting soil nematicidal capacity [18]. Finally, sulla is also an important honey plant [19]. The global concern regarding the decline in wild pollinators will possibly further enhance its cultivation in Europe as part of the EU strategies to reverse this dangerous trend by 2030 (e.g., EU biodiversity strategy for 2030 and the EU Pollinators Initiative). Due to the crop’s multifunctional value, there is a renewed interest also for breeding: in Australia, a newly released variety named ‘Flamenco’, suitable for low- and medium-rainfall areas, was specifically improved for seed production and dehulling [20]. Under the current scenarios of climate change and water scarcity, it is important to evaluate sulla drought tolerance and find novel genetic resources useful for breeding to enhance the competitiveness of this crop in Mediterranean countries. Substantial genetic variability exists in natural populations, and this supports the search of potential donors for breeding programs [21]. Responses to drought stress have been evaluated for a subset of Italian and Tunisian commercial varieties and ecotypes grown at multiple locations with contrasting rainwater availability [22]. Some materials have proven to be specifically adapted to high or low rainwater availability: the Italian variety Sparacia performed poorly in cold, wet environments but showed greater adaptability to water-limited conditions (annual precipitation < 500 mm); two ecotypes of central Italy have shown wide adaptability to both water-favorable and water-limited environments and have outperformed many commercial varieties under moderate drought stress. Although the large majority of drought stress tolerance research has been focused on above-ground morphological and physiological parameters, there is a growing interest on root traits [23,24]; and architectural traits such as root length, diameter, and maximum rooting depth are correlated with water acquisition efficiency and hence with drought avoidance [25,26,27,28]. To the best of our knowledge, no data exist on the effect of drought stress on sulla forage quality and root growth: drought sensitivity in sulla has so far been evaluated only in terms of forage yield and only on mature plants. Plants in early vegetative stages are very sensitive to both abiotic and biotic stress due to their small photosynthetic canopy. Early drought stress tolerance is especially important for forages because grazing and/or clipping systematically sets back the crop ontogenetic stage.

The aim of our work was to evaluate the effect of moderate drought stress (50% of field capacity) on the shoot biomass and architecture, forage quality, and root length density of sulla during the early vegetative growth stage. We compared a commercial variety widely cultivated in Italy, the cv. Bellante, and a newly bred Italian variety named Centauro, registered in 2014 in the Italian national register of plant varieties but not yet available on the market—under the hypothesis that drought stress would affect both above and below-ground traits with possible interactions between variety and moisture availability. Both varieties were strongly affected by drought stress without significant differences between each other. Constitutive differences between cultivars emerged across water levels. Centauro developed more shoots and leaves than Bellante and also more root length: these traits point toward a higher potential forage palatability (shoot traits) and to a better foraging capacity (root traits) that is beneficial in low-input systems.

## 2. Results

### 2.1. Shoot and Root Growth

**Figure 1:**
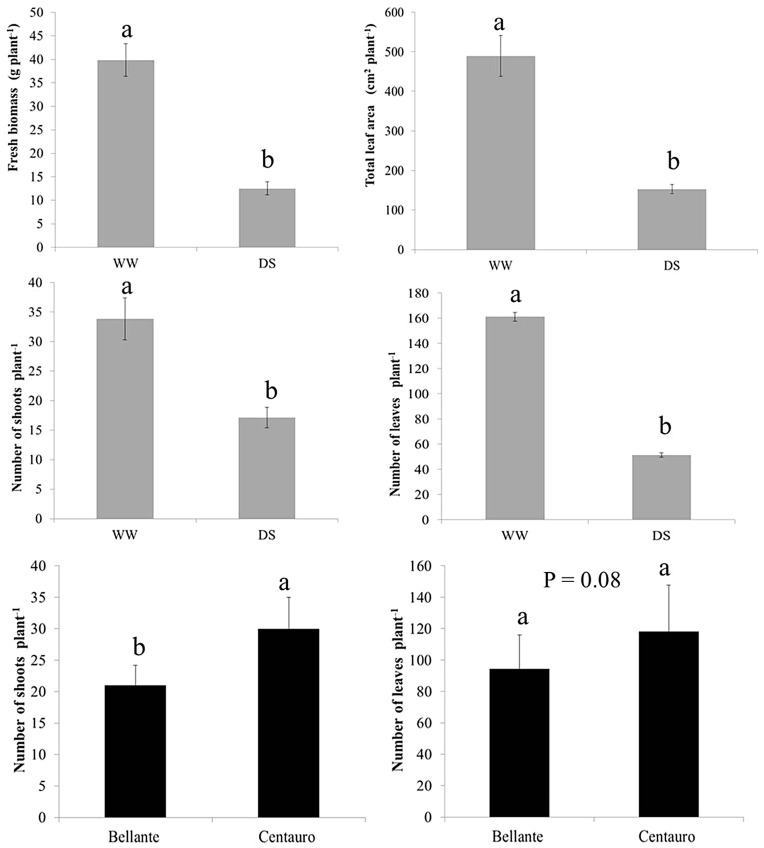
Shoot traits as affected by drought stress (grey solid columns) and variety (black solid columns. Bar plots of the means (average across varieties and drought stress) are overlaid by the standard error of the mean bars. Different letters (a and b) above the bars indicate significant differences (0.95%) among the means.

Summary statistics of root and shoot biometry are reported in Table 1 and the ANOVA results are reported in Table 2. Bar plots of the mean values of shoot and root parameters are depicted in Figure 1.

Under optimal irrigation, both varieties produced a large number of shoots and leaves and a high leaf area. Also, a relatively high total root length density was produced (2.3 cm cm^−3^ average across genotypes), 56% of which was found in topsoil (0–0.15 cm surface layer). Shovel roots were found without significant differences between treatments, whereas nodulation was almost absent.

Plants sizes varied substantially within each variety under both water regimens, so biomass, shoot length, and leaf area differences between varieties were not significant.

Constitutional differences between varieties emerged for shoot number, which was significantly higher for Centauro than Bellante across water regimes (+46% *P* < 0.05). There was also a tendency for a greater leafiness (+26% total leaf number, *P* = 0.08), a trait possibly associated with a superior palatability.

**Figure 2:**
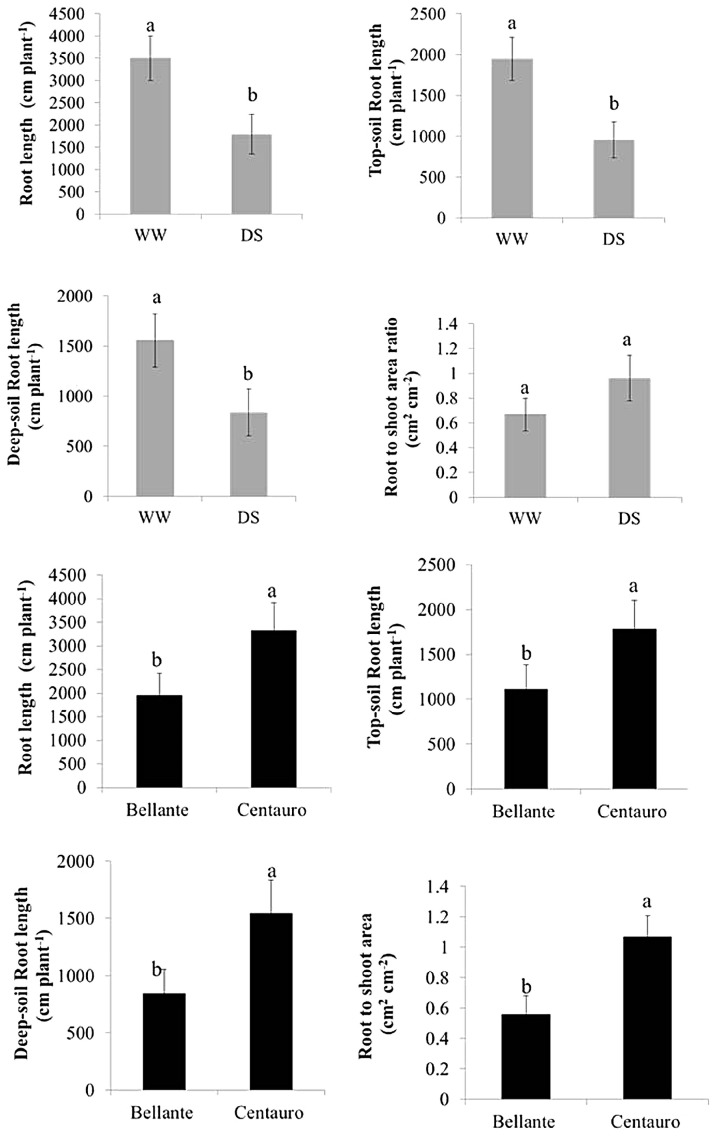
Root traits as affected by drought stress (grey solid columns) and variety (black solid columns. Bar plots of the means (average across varieties and drought stress) are overlaid by the standard error of the mean bars. Different letters (a and b) above the bars indicate significant differences (0.95%) among the means.

Drought stress severely impacted both shoot (Figure 1) and root traits (Figure 2). As shown in Figure 1, the most drastic response to drought stress occurred in the aboveground compartment with a drastic reduction in the number of leaves and leaf area and a severe reduction in fresh biomass. Differences between varieties in shoots and leaves are only significant for shoot number, which are more evident in well-watered conditions.

**Figure 3:**
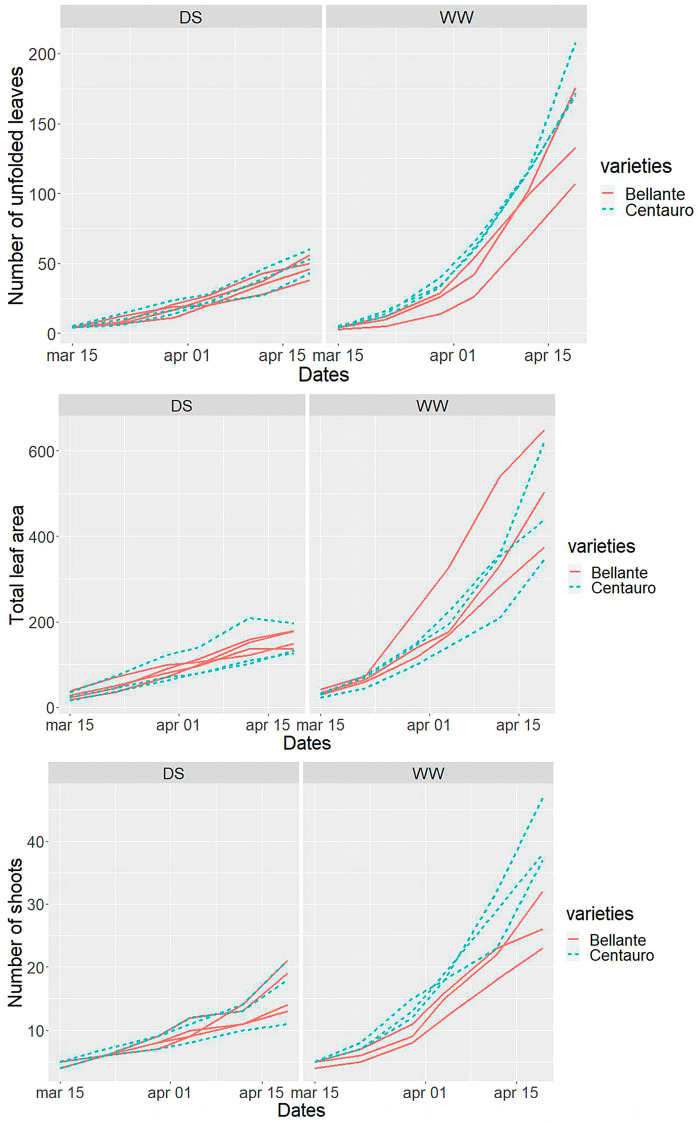
Line plots (from left to right) of the total number of unfolded leaves, the total leaf area, and the number of shoots for Bellante (solid red line) and Centauro (dashed blue line) under drought stress (DS) and well-watered (WW) conditions.

The two varieties differed mostly for root architecture (Figure 2, black columns): Centauro displayed a significantly higher total root length (+70% *P* = 0.04) with more roots than Bellante both in the topsoil (+60% *P* = 0.05) and in the deeper strata (+80% *P* = 0.04). A larger total root-projected area was also found in Centauro (+90% *P* < 0.01), +83% in the surface soil (*P* < 0.01) and +108% in the bottom layer (*P* = 0.01) (average across water regimes). Centauro tends to invest more in roots than in shoots (*P* = 0.08). Both varieties were severely affected by drought stress, and no significant genotype × environment interaction was found. The fresh biomass was reduced to nearly 1/3 (−69% *P* < 0.05) and so were the total number of leaves (−68% *P* < 0.05), shoots (−49% *P* < 0.05), and leaf area (−69% *P* < 0.05). Drought stress halved the root length (−49% *P* < 0.05) slightly more in topsoil (−52%) than in deeper soil (−46%) but did not significantly modify the partitioning between shoots and roots (*P* > 0.05). The root-area-to-leaf-area ratio was significantly higher in Centauro (*P* = 0.02), about two times that of Bellante under both water-favorable and drought-stressed conditions. This greater root investment for Centauro did not result in a higher yield or a better drought tolerance but, at the same time, was not a disadvantage in terms of resources allocated to the canopy. As mentioned, no significant interaction was found for shoot and root traits but there was a trend in the data indicating that the yield penalty caused by drought tends to be lower for Centauro for fresh biomass (−62% Centauro and −75% Bellante) and for leaf area (−66% vs. −71% Centauro and Bellante, respectively). Similarly, the below-ground yield penalty was somehow lower for Centauro. In drought-stressed plants, root length was reduced by −47% in Centauro and −52% in Bellante, and root area decreased by 50% in Centauro and by 56% in Bellante. Drought stress also affected growth rate. The shoot growth curve is depicted in Figure 3. There is a large scattering in the data but drought stress clearly reduced and slowed down shoot growth rate.

The line plots show that under well-watered conditions, Centauro growth curves overtop those of Bellante for the total number of leaves and shoots but not for the total leaf area, indicating that Bellante develops fewer but larger leaves. Growth curves under drought stress show substantial overlap. Under drought stress, shoot parameters increase linearly in time, while under optimal irrigation, all shoot parameters follow an exponential growth. To test the experimental factors on the shape of the growth curves, the data were analyzed within the framework of the Generalized Additive Models (GAMs). The GAM was computed on the median values of the combination of irrigation level and variety at the measurement dates. GAM allowed the testing of both the quantitative effect of genotype and irrigation levels (model fixed terms) as well as the effects of irrigation on the shape of the growth curves (smoothing terms). Model summery statistics are reported in Table 3.

**Figure 4:**
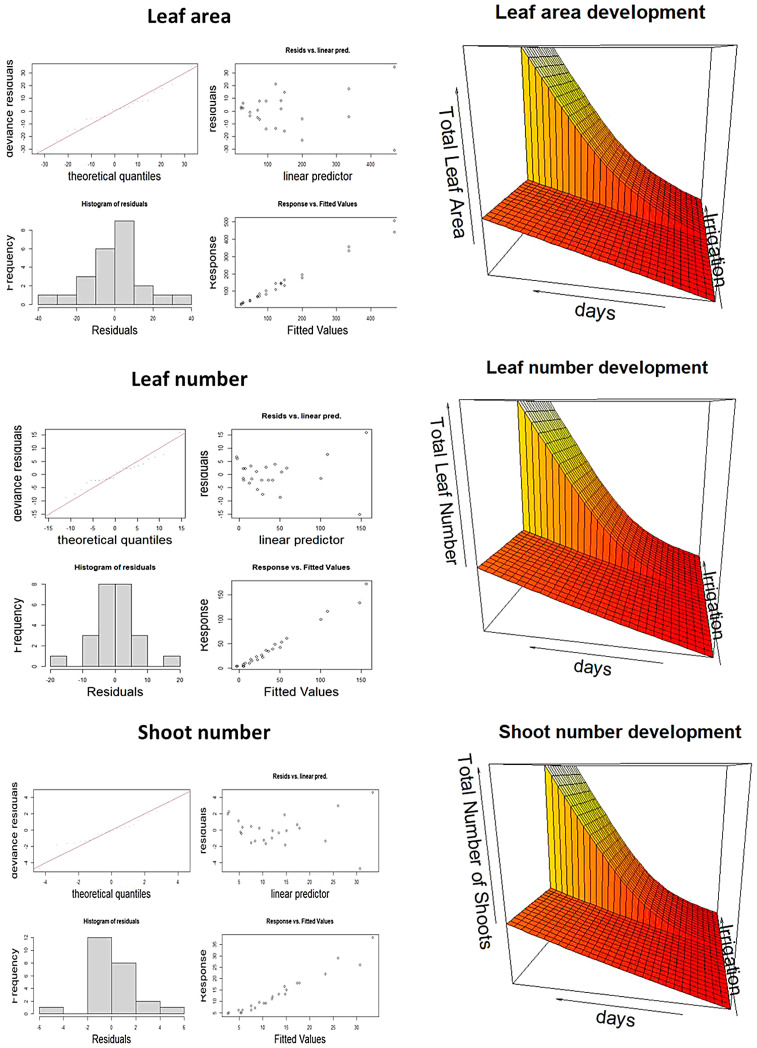
GAM model result. (**left**) Graphical analysis of the goodness of fit and (**right**) the trend surface for the three shoot parameters (number of leaves, leaf area, and number of shoots).

The goodness of fit was evaluated both through the proportion of explained variance and through graphical analyses (Figure 4, left side).

For all shoot parameters, irrigation is significant as a fixed effect (indicating that the plants grow significantly more under non-limiting water), and the significant smooth terms indicate that there is also a significant difference between the shape of curves. Under drought stress, the low effective degrees of freedom (edf) close to 1 indicate a linear pattern while larger edf values in the well-watered treatment indicate a non-linear growth. The trend-surface for the three shoot parameters (number of leaves, leaf area, and number of shoots) are depicted on the right side of Figure 4. For the number of leaves and shoots, Centauro growth curves overtop those of Bellante under well-watered conditions but the shape of the curves does not vary between varieties: the variety was never significant as a smooth term. Both the high percentage of deviance explained (always >90%) and the plot of the predicted vs. observed data indicate that the models capture a substantial portion of variability in the data.

### 2.2. Forage Quality

Mean values and the standard deviation of quality parameters for each variety × irrigation level combination are reported in Table 4.

Extractable and protein-bound CT had the largest fractions of condensed tannins. ANOVA results are reported in Table 5. No significant differences between varieties were found, but both extractable CT and total CT showed a significant variety × irrigation interaction.

Under optimal irrigation, Centauro was less tanniferous than Bellante (−35%) but the opposite occurred under drought stress, under which Centauro produced 50% more CTs than Bellante. Centauro markedly increased extractable tannins under drought stress (+81%) while the opposite occurred in Bellante (−20%). Nitrogen content was significantly reduced by drought and differences amounted to 12% on average across genotypes. Neutral detergent fiber (NDF) and total polyphenol content showed no significant variations between genotypes and irrigation levels. The low variability in NDF could be due to the juvenile stage and the short duration of the drought stress, which prevented tissue lignification. Polyphenol content was generally high but showed substantial variability within treatments, especially in well-watered Bellante (38% CV).

## 3. Discussion

### 3.1. Shoot and Root Growth

In our experiment, sulla proved to be sensitive to drought stress as even the moderate stress level tested in our case more than halved most shoot parameters and also severely reduced the root length both in commercial and newly bred material. Lower yield penalties under stress (from 8% to 24%) were reported for *Trifolium repens* L. and *T. pratense* L., while yield reductions from 51% to 68% were reported for *Lolium perenne* L. and *Cichorium intybus* L. [29]. Multiple drought stress events reduced the forage yield of minor legumes *Medicago lupulina* and *Lotus corniculatus* vs. (−26% DM on average) and penalized *Trifolium repens* (−43%) to a greater extent [30]. Lower yield penalties were reported for alfalfa subjected to a moderate–severe spring drought stress (10–15% of usable water capacity), and yield penalties ranged from 8% to 13%, respectively [31]. In Liu et al. [31], however, soil moisture was measured in the first 1.5 m, but alfalfa roots can penetrate deeper in the profile and withdraw water from deeper strata. The higher yield penalties in our experiment may also be due to the early, juvenile stage. Drought sensitivity is affected by growth stage: across a wide range of soils and climate, drought-stressed maize yields were closely associated with the accumulation of biomass [32]. A greater drought sensitivity during juvenile stages was found by Malisch et al. [33] in Sainfoin (*Onobrychis viciifolia*): drought stress reduced biomass by 56% during the vegetative stage, while the drought-related yield penalty was negligible (2%) during the reproductive stage. In our data, sulla root length was also reduced by drought stress though slightly less than the above-ground counterpart. The reduction in root density in response to drought stress is part of an adaptation mechanism (similar to the reduction in leaf area) to avoid water over-use. Under drought stress, several grasses suppress nodal root growth; this mechanism was found in the C4 model plant *Setaria viridis*, sorghum (*Sorghum bicolor*), switchgrass (*Panicum virgatum*), and *Brachypodium distachyon* and the wild ancestor of maize (teosinte). This was interpreted as an adaptive mechanism to prevent excessive water uptake during the vegetative stage [34]. In some cases, a compensatory growth of primary root axes improved drought resistance: maize lines with few but long laterals conserved a better water status under limited water availability and yielded up to 144% more than a contrasting phenotype with dense but shorter laterals [35]. Interestingly, the authors showed that these two contrasting phenotypes showed similar values of total length density under both well-watered and water-limited conditions, where root length was halved in both genotypes. In such cases, only root topology may reveal different adaptation mechanisms.

In our experiment, cv. Centauro showed a constitutively high root length and projected area, but this did not result in a greater drought tolerance, since the yield penalty for this cultivar was smaller but non-significant. Large root systems have been interpreted both as a drought-tolerance trait and as a characteristic associated with well-watered environments. Zhang et al. [36] showed that the drought-tolerant alfalfa cv. Longzhong exhibited a greater root biomass and root-to-shoot ratio. This trait is beneficial if roots can access distant or deep water reserves. A vigorous root system, however, can also deplete soil water reserves too rapidly during the season, as discussed for wheat in Mediterranean climates [37]. Kang et al. [38] found that the alfalfa cultivar Mamuntanas exhibited a lower early root and shoot growth, and this conferred a higher drought tolerance to this genotype, possibly associated with a parsimonious use of water; such a trait is beneficial if the soil water reserve is truly limited and not just out of reach for small rooted plants. Similarly, a maize genotype mutant that does not form nodal roots maintained a better water status, avoiding water over-use [35]. Water consumption, however, is not just a function of root length but also depends on root hydraulic conductivity. The importance of root anatomy in reducing water use before anthesis has been elucidated for wheat by comparing genotypes selected for a reduced vessel diameter [39]: individuals with narrow vessel diameters (reduced from 66 mm to less than 56 mm) yielded between 3 and 11% more than the unselected controls, and had a higher harvest index, biomass at maturity, and kernel number. In our experiment, the larger root length density of Centauro did not result in a faster depletion rate, as the growth curves of the two varieties overlapped, showing no sign of earlier stress in Centauro. This may point toward a better stomatal control or changes in root hydraulic conductivity that may have slowed down depletion; these hypotheses, however, need to be tested with further research. The role of a large root length in Centauro in drought-prone environments will depend on the specific agro-environment and management.

Lynch [40] argues that in high-input yet water-limited environments, a root phenotype characterized by fewer axial roots and a reduced density of lateral roots is more efficient: the metabolic cost of a dense root system is not required, since nutrients are not limited and the parsimonious water use of sparse roots translates into a ‘water banking strategy’ to be used later in the season. Conversely, in low-input environments, a dense root system (aggressive phenotype) competes better for nutrients and space occupancy and would also be more responsive to transient nutrient availability or to sparse rain events [40]. The higher root length and area of Centauro may confer to this cultivar a greater responsiveness to in-season precipitation since roots can capture water before evaporation depletes it: a modeling study aimed at comparing the transpiration of plants with a contrasting root architecture under different drought scenarios has shown that plants with a high root density in surface layers are very efficient in the so-called ‘supply driven environments’ in which water is supplied through in-season precipitation and roots must absorb it before it is lost through evaporation [41]. A larger root density may also confer a superior competitive ability in mixed stands and in cases of reduced or null herbicide availability (e.g., organic systems).

Constitutive traits are associated with high heritability and this is important for breeding. As pointed out by Wasson et al., 2012 [42], root traits tend to have a low heritability and high plasticity, which favors adaptation to highly variable environmental conditions (e.g., soil type and rain pattern), but this is counterproductive for breeding and is the reason why plant breeders tend to recoil from selection based on root traits. The constitutive high root density of Centauro may be further tested for heritability and used in breeding programs aimed at improving the root sink. In our pots, nodulation was almost absent; this was due to the absence of *rhizobium hedysari*—the root nodule microsymbiont specific for sulla—in the field soil used for the experiment of natural sources. Both varieties developed the unique-species, modified lateral root structures called ‘shovel roots’: peculiar curved flat shaped roots that resemble a shovel. Shovel roots are known to be calcium-absorbing organs; by removing the carbonate buffering in soils, they render the rhizosphere acidification more efficient and hence facilitate Fe and P uptake in alkaline soils [43]. Due to their short, flat shape, shovel roots have a limited impact on root length but increase the root surface. In our work, the number of shovel roots was highly variable and did not show significant relationships with shoot traits; however, the root-projected area (which is affected by the presence of shovel roots) was strongly correlated with fresh biomass in drought stress treatments. Also, in drought stress, only a linear trend appears (R^2^ = 0.56) between the number of shovel roots and shoot fresh biomass. The low number of observations available in our study does not allow for inference through correlation analysis and the trends in the data remain just indicative. These preliminary observations, however, suggest that the role of shovel roots in drought stress tolerance may deserve investigation. Finally, sulla is considered a soil ameliorative plant. Due to its higher root length variety, Centauro may be better suited for land amelioration than Bellante: root length density is in fact positively correlated to soil reinforcement by roots [44] and also for C-farming as roots are the major C producer in soils [45]. More research on sulla roots is therefore needed in the direction of yield-related and environmental roles, and caution needs to be used when comparing data across experiments especially for root length: for other species, values have been reported to vary greatly with genotype, growth environment, and phenology. Values reported in our experiments are within the range of magnitudes of legume plants grown for a few weeks in lab settings where, for instance, the total root length has been reported to vary between 305 and 3824 cm for chickpea genotypes [46]. Other authors [47] have also shown that different methods of root washing before measurement are responsible for considerable differences in the loss of fine roots. This methodological issue has been considered large enough to explain literature discrepancies of two orders of magnitude in root length values but not in root mass [47].

In agreement with data on growth rates of forage crops under drought stress, leaf appearance and tillering were reduced and slowed down by drought. In ryegrass, drought resulted in a slower leaf expansion and leaf appearance [48,49,50]. In soybean, drought stress caused a transient reduction in the growth rate (number and length of nodes and leaf area), which, however, was recovered after rains [51]. These morphological changes are adaptive responses to water shortage, since they would reduce the radiation load on leaves and transpiration. In our experiment for drought-stressed plants, all shoot traits increase linearly in time and differences between varieties are flattened. Under optimal irrigation, shoot growth is exponential and Centauro overtops Bellante for the number of leaves, a characteristic possibly predictable of a greater palatability.

### 3.2. Forage Quality

Condensed tannins (CTs), also known as proanthocyanidins, are complex molecules involved in plant defense against pathogenic micro-organisms and herbivores [52]; they also exert a role in abiotic stress tolerance protecting plants from oxidative stress [53]. CT content variation is important for feed quality, and above a certain threshold, tannins exert anti-nutritional effects by reducing forage palatability. Values around 55 g CT kg DM in big trefoil (*Lotus pedunculatus*) reduced sheep’s feed voluntary intake by 12% [54]. The latter dropped to −27% with a CT concentration between 63 and 106 g/kg DM. Sulla in our trial displayed a moderate CT content with peak values of 5% extractable CT in drought-stressed plants; values of both varieties are within the range for this crop (2–12% DM [52], 3.3% DM [55]). Our values are within the threshold of non-reduced palatability: a moderate CT content in sulla (between 40 and 50 g/kg DM) has been shown to not depress voluntary intake in lambs, and the associated high ratio of readily fermentable structural carbohydrates increased the growth rate as compared to low-CT pastures only [56]. Even in case of a higher CT concentration of 7.2%, sulla forage proved to be highly palatable for young sheep, with a high digestibility (70% DM digestibility) and doubled fecal N content; this reduced the urinary output and therefore the potential of N leaching to groundwater [57]. The anthelmintic effects of sulla forage have been reported by several researchers [10,58] even with a CT concentration lower than the values reported in this work (1.7–2%) [59]. Condensed tannins’ indirect (immunologically/physiologically mediated) effects on host responsiveness against gastric nematodes have been reported for sulla [60]: lambs grazing sulla with a CT content of 36.9 g kg DM showed elevated immune responses against *T. circumcincta* infections, and this was ascribed to the capacity of CT to bind dietary proteins, thereby protecting them from rumen degradation. An extra protein supply in turn would stimulate the immune system against gastro-intestinal parasites. Our work shows that CT content is increased through drought stress; a similar response was also found in *Casuarina equisetifolia* seedlings and discussed as an adaptive trait: the increase in tannin content in drought-stressed plants would help prevent water losses by reducing the cell water potential [61]. Condensed tannins are powerful antioxidants and may help neutralize reactive oxygen species that accumulate under drought stress. A CT content upsurge was measured in drought-stressed poplar [62] and eucalyptus [63]. Condensed tannin levels in trefoil (*Lotus corniculatus* L.) also increased during periods of drought stress [64]. An interesting study by Malisch et al. [33] shows that CT response to abiotic stress is affected by the ontogenetic stage: sainfoin showed a marked response to drought stress during the vegetative stage when leaf CT concentration raised up to 46%, whereas during the reproductive stage, the impact of drought stress on growth was negligible and leaf CT concentration was reduced by 9%. In our experiment, protein-bound and fiber-bound CTs were low and stable across genotypes and irrigation status. This is in agreement with Malisch et al. [65] who found that sainfoin-bound CT was low and stable across genotypes, growth stages, and drought levels and thus was considered as having a low impact on feeding value. Studying plant abiotic stress tolerance during the early vegetative stage is very important for forage crops, not only because young plants are especially vulnerable to shoot damages due to their relatively small photosynthetic canopy but also because grazing or cutting systematically sets back the ontogenetic stage to the early vegetative stage several times during the life of a forage crop. Our results show that under both well-watered and drought-stressed conditions, CT levels are within a range tolerable for voluntary intake; still, based on the literature, anthelmintic effects can be expected. If CT biosynthesis is the target, then drought stress can be used to enhance forage anthelmintic capacity. The higher plasticity displayed by Centauro suggests that this variety may specifically modulate CT biosynthesis as part of the drought stress tolerance strategy. The tendency for lower CT content under well-watered conditions coupled with the higher leafiness may also suggest a higher palatability of this cultivar.

## 4. Materials and Methods

### 4.1. Experimental Setup

Two experimental factors with two levels each were tested in factorial combination with three replications:

- Factor 1. Genotypes of sulla (*Hedysarum coronarium* L.) with two levels:

. Variety Bellante (kindly supplied by Padana Sementi srl).

The newly released variety named Centauro registered in the Italian national register of plant varieties in 2014 (decree of the Italian Ministry of Agriculture n. 5750 of 13 March 2014) (https://www.sian.it/mivmPubb/listeVarieta.do, last access 4 September 2023) but not yet available on the market. Seeds of Centauro were supplied by the CREA ZA germplasm collection. Factor 2. Water stress conditions with two levels:

. WW: well-watered—irrigated to 80% field capacity (FC) throughout the experiment. DS: drought-stressed—irrigated to 80% field capacity for 4 weeks (when about 20 leaves were developed). Afterward, soil water content was allowed to decrease to 50% FC, and pots were thereafter irrigated to maintain 50% FC until the end of the experiment.

The 80% FC level was chosen as appropriate for well-watered conditions as commonly performed (e.g., for legumes [66]) in order to avoid possible waterlogging spots, whereas values of 40 to 50% are commonly identified as appropriate for definite water stress conditions (e.g., for legumes [66]).

Cylindrical pots (8 cm diameter, 30 cm tall) were filled with agricultural loamy soil (47% sand, 39% silt, 24% clay) with pH 7.3, C 2.05%, N 0.21%, extractable P 77 mg kg^−1^, and exchangeable K 1755 mg kg^−1^. Three vertical holes were dug in the soil of each pot from top to bottom in order to ensure a uniform water distribution, the soil was initially brought to field capacity, and irrigation was applied twice a week after water losses were determined gravimetrically.

Seeds were pre-germinated in the dark for 3 days, transplanted with a radicle of about 0.5 cm, and grown indoors for 9 weeks under artificial lighting (Osram fluora at 400 mmol m^−2^ s^−1^) at room temperature (22 °C on average) under a progressively increasing photoperiod from 11 to 12 h of light. Pots were arranged following a completely randomized experimental design.

### 4.2. Shoot Biometry

Starting from the date when the water stress treatments were differentiated (4 weeks after sowing), the following shoot traits were determined biweekly: number of shoots, maximum shoot length (cm), and number of unfolded leaves. With the aid of a ruler, the length (l) and width (w) of individual leaves were measured. Approximate individual leaf area was calculated as

Individual leaf area = l × w cm^2^

The area of individual leaves per plant was summed up to obtain approximate total plant leaf area. At harvest, shoot fresh biomass was determined on the whole harvested plant. Shoot dry matter was measured after oven drying at 70 °C for 72 h on a subset of shoots while the rest were devoted to forage quality analyses.

### 4.3. Forage Quality

Forage qualitative analysis was conducted on fresh above-ground biomass collected at harvest and preserved under vacuum at −80 °C. Chemical analysis was carried out in service by Servizio Analisi conto terzi UNIPG DSA3-UR Chimica Agraria. Specifically, each whole-plant (stem and leaves) sample was freeze-dried and ground in a large Wiley mill to pass a 1 mm screen. The following parameters were determined analytically: nitrogen content (N) (Kjeldahl method), neutral detergent fiber (NDF) [67], and total polyphenols using Folin–Ciocalteu reagent with the protocol reported by [68]. Crude protein content was calculated as

Crude protein = N × 6.25.

Condensed tannins (CTs) were determined through a procedure whereby three CT fractions are extracted and quantified: free or extractable, protein-bound, and fiber-bound CT [57]. For each replication, two 500 mg lab duplicate samples of freeze-dried milled material were weighed into 50 mL screw-top polyallomer centrifuge tubes. On each sample, free CP were extracted with a mixture of acetone/water/diethyl ether (4.7:2.0:3.3 v) [55], which allows the extraction of free condensed tannins and the wash out of lipids and non-tannin pigments all in one step. On the solid residue of this extraction, protein-bound CT was separated with boiling sodium dodecyl sulphate containing 2-mercaptoethanol (SDS); subsequently, the fiber-bound CT fraction was determined directly on the residue from protein-bound CT extraction, through the addition of 30 mL of butanol/HCl and 3 mL of SDS solution.

### 4.4. Root Traits

For root length determination, pots were stored at −20 °C for a few weeks. Frozen pots were then split into two halves of 15 cm each (top and bottom) with the aid of a circular saw. Pot halves were unfrozen overnight in water and sodium hexametaphosphate (15 g/L) and were than elutriated over stacked soil sieves from 2 mm to 0.4 mm. Roots collected from all sieves were stored in ethanol (50% *V*/*V*) at 4 °C until scanning and image analysis. The image analysis software WinRhizo ArabidopsisV2009c (Regent Instruments Inc., Quebec, Canada) was used to quantify root length (RL, cm) and root-projected area (cm^2^) separately on the top and bottom pot halves, and the values were, respectively, labeled as Top-Root Length and Area and Deep-Root Length and Area.

On each image, visible shovel roots were counted manually to give the total number of shovel roots per plant. Plants were evaluated for nodulation, and since there was almost no nodulation (0–3 nodules per plant), data were not analyzed.

### 4.5. Statistical Analysis

Shoot and root morphological traits and forage quality were analyzed through a 2-way ANOVA followed by Tukey’s post hoc tests to find significant differences between treatments and their interactions (*P* < 0.05). To test treatments effects on the shoot growth pattern, the time series of shoot measurements were analyzed within the framework of the Generalized Additive Models (GAMs), which a flexible generalization of linear models suitable for modeling both linear terms and non-linear terms through the estimate of parametric coefficients and the use of non-parametric or semi-parametric smoothing functions. The GAM describes the relationship between the continuous response variable, Y, and several predictor variables, ‘xi’. A normal distribution from the exponential family was chosen to represent Y, within an identity link function (g) that links the expected value of E(Y) to the predictor variables through a structure (fi) that can take a linear form (parametric terms) of a non-parametric or semi-parametric specification using ‘smooth functions’ (splines, ‘s’) alone or in interaction with fixed categorical factors [69]. The basic form of a GAM model is the following:

*g*(E(Y)) = *β*_0_ + *f*_1_(*x*_1_) + *f*_2_(*x*_2_) +……+ *f_m_*(*x_m_*).

In order to test whether the shape of the growth curve for each shoot parameter was affected by irrigation, the response variable ‘Y’ was modeled as a function of the parametric terms (irrigation and variety), and a smoothing function (s) was used to model the effect of time (days) in interaction with irrigation:

Y = β₀ + β_1_ irrigation treatment + β_2_ varieties + s (days, k = 6, by = treatment)

GAMs were fitted using penalized likelihood maximization, and smoothing parameters were automatically chosen to minimize the Generalized Cross-Validation criterion. Models were checked for violation of independence, variance homogeneity, and residual normality by graphical outputs. GAM was computed using the ‘mgcv’ library [70] within the R statistical environment (R Core Team (2022). R: A language and environment for statistical computing. R Foundation for Statistical Computing, Vienna, Austria. URL https://www.R-project.org/ (accessed on 10 August 2023)).

## 5. Conclusions

This preliminary work provides the first contribution on the qualitative and quantitative early response of sulla shoot and rootsto drought stress. The modern germplasm of sulla tested in this work, both the newly bred variety Centauro and the widespread commercial variety Bellante, was sensitive to drought stress: above and below-ground growth was halved when soil moisture content was reduced to50% of field capacity. Both varieties therefore proved to be more suitable for well-watered environments. The variety Centauro did not outperform the commercial variety Bellante in terms of biomass production and did not show a superior drought tolerance. Due to the large variability among individuals, differences between varieties were seldom significant, and in order to be fully representative of productive performances, varieties should be evaluated in field settings over multiple years and on a number of replicates sufficient to encompass the inherent variation due to allogamy. Even under the limited lab experimental setting, in our work, the variety Centauro showed some morphological differences such as a significantly larger number of shoots and a tendency for a higher leafiness especially under well-watered conditions. Also, in favorable water conditions, the variety Centauro produced less CTs than the variety Bellante. These parameters may positively affect forage palatability and animal voluntary intake and thus point toward a greater grazing value for this cultivar. Under drought stress, forage yield was markedly reduced and the quality was affected with a reduction in crude protein content and an increase in extractable CT. As CTs affect both palatability and anthelmintic properties, it is important to take into account plant water status for forage quality evaluation. Centauro showed a greater plasticity in CT production in response to water availability, suggesting that tannin production may retain an adaptive value for this cultivar. Centauro displayed a higher root length and root-projected area, traits generally associated with a superior foraging capacity and competitiveness, and thus useful in low input environments. Also, a proliferative root system can be more responsive to in-season precipitation, thereby increasing plant drought tolerance in water-limited, supply-driven environments. This variety may therefore be further investigated as a potential donor in breeding programs specifically focused on root traits.

## Figures and Tables

**Table 1:** Mean, standard deviation, and coefficient of variation (CV%) of shoot and root morphological parameters under well-watered (WW) and drought stress (DS) conditions.

		Mean														
Irrigation	Variety	Fresh Biomass Plant^−1^ (g)	Dry Biomass Plant^−1^ (g)	Dry Matter %	N° of Shoots	N° of Leaves	Max Shoot Length (cm)	Leaf Area (cm^2^)	Root Length (cm) Plant^−1^	Top- Root Length (cm) Plant^−1^	Deep- Root Length (cm) Plant^−1^	Root Area (cm^2^) Plant^−1^	Top- Root Area (cm^2^) Plant^−1^	Deep- Root Area (cm^2^) Plant^−1^	N° Shovel Roots Plant^−1^	Root-to-Shoot-Area Ratio
WW	Bellante	41.5	3.49	8.38	27	139	16.3	510	2645	1542	1103	217	126	90.8	61.3	0.43
	Centauro	38.2	3.35	8.77	40.7	183	13.8	468	4354	2345	2009	403	219	184	50.3	0.907
DS	Bellante	10.6	1.18	11	15	49.7	10.8	147	1272	681	592	95.4	56.4	39	34.7	0.691
	Centauro	14.4	1.74	11.7	19.3	53	12.3	159	2310	1229	1081	200	114	85.3	52.7	1.23
		Standard Deviation														
WW	Bellante	12.2	1.07	0.206	4.58	34.8	3.4	137	1206	722	539	96.7	52.6	49.3	7.77	0.18
	Centauro	5.15	0.53	0.41	5.51	21.4	3.62	140	216	191	376	31.5	12.1	36.5	39.7	0.238
DS	Bellante	0.678	0.155	0.839	5.29	6.51	1.04	28	479	142	393	36.5	12.8	25.7	29.7	0.364
	Centauro	4.38	0.814	2.06	1.53	7	3.01	33.4	1394	701	698	91.1	52	40.8	33.5	0.39
		Coefficient of variation %														
WW	Bellante	29.4	30.66	2.46	16.96	25.04	20.86	26.86	45.6	46.82	48.87	44.56	41.75	54.3	12.68	41.86
	Centauro	13.48	15.82	4.68	13.54	11.69	26.23	29.91	4.96	8.14	18.72	7.82	5.53	19.84	78.93	26.24
DS	Bellante	6.4	13.14	7.63	35.27	13.1	9.63	19.05	37.66	20.85	66.39	38.26	22.7	65.9	85.59	52.68
	Centauro	30.42	46.78	17.61	7.93	13.21	24.47	21.01	60.35	57.04	64.57	45.55	45.61	47.83	63.57	31.71

**Table 2:** ANOVA results. Parameters with *P* > 0.05 is non significant (ns).

	Drought Stress	Variety	Drought Stress × Variety
**N° of leaves**	*P* = 1.7 × 10^−5^	*P =* 0.08 ns	ns
**N° of shoots**	*P* = 0.008696	*P* = 0.000212	ns
**Fresh Biomass plant^−1^ (g)**	*P* = 0.000142	ns	ns
**Dry Biomass plant^−1^ (g)**	*P* = 0.00156	ns	ns
**Max shoot length (cm)**	*P* = 0.074 ns	ns	ns
**Leaf area (cm^2^)**	*P* = 0.000408	ns	ns
**Root Length (cm) plant^−1^**	*P* = 0.0149	*P* = 0.038	ns
**Top-Root Length (cm) plant^−1^**	*P* = 0.0106	*P* = 0.0533	ns
**Deep-Root Length (cm) plant^−1^**	*P* = 0.0427	*P* = 0.048	ns
**Root Area (cm^2^) plant^−1^**	*P* = 0.00408	*P* = 0.00739	ns
**Top-Root Area (cm^2^) plant^−1^**	*P* = 0.0042	*P* = 0.00889	ns
**Deep-Root Area (cm^2^) plant^−1^**	*P* = 0.0101	*P* = 0.0145	ns
**Root-to-Shoot-Area Ratio**	ns	*P* = 0.0205	ns
**N° Shovel root plant^−1^**	ns	ns	ns

**Table 3:** GAM model summary statistics. For all the computed models, a Gaussian family distribution and an identity link function from top to bottom were assumed.

Family: Gaussian-Link function: identity				
**Leaf Area**~treatment + s (days, k = 6, by = treatment)				
Parametric coefficients:				
	Estimate	Std.Error	t value	Pr(>|t|)
(Intercept)	85.664	4.662	18.37	3.33 × 10^−13^ ***
**treatmentWW**	**121.307**	**6.593**	**18.40**	3.25 × 10^−13^ ***
Approximate significance of smooth terms:				
	edf	Ref.df	F	*p*-value
**s (days): treatmentDS**	**1.00**	**1.000**	**83.3**	<2 × 10^−16^ ***
**s (days): treatmentWW**	**2.79**	**3.397**	**319.6**	<2 × 10^−16^ ***
Signif. codes: 0 ‘***’ 0.001 ‘**’ 0.01 ‘*’ 0.05 ‘.’ 0.1 ‘ ’ 1				
R-sq.(adj) = 0.985; Deviance explained = 98.8%				
GCV = 343.78; Scale est. = 260.84; n = 24				
**Leaf Number**~treatment + varieties + s (days, k = 6, by = treatment)				
Parametric coefficients:				
	Estimate	Std. Error	t value	Pr(>|t|)
(Intercept)	19.417	2.537	7.652	7.15 × 10^−7^ ***
**treatmentWW**	**36.167**	**2.930**	**12.344**	**7.50 × 10^−10^** ***
**varietiesCentauro**	**8.000**	**2.930**	**2.730**	0.0143 *
Approximate significance of smooth terms:				
	edf	Ref.df	F	*p*-value
**s (days): treatmentDS**	**1.452**	**1.763**	**35.75**	2.66 × 10^−6^ ***
**s (days): treatmentWW**	**2.740**	**3.339**	**198.50**	<2 × 10^−16^ ***
**---**				
Signif. codes: 0 ‘***’ 0.001 ‘**’ 0.01 ‘*’ 0.05 ‘.’ 0.1 ‘ ’ 1				
R-sq.(adj) = 0.975; Deviance explained = 98.1%				
GCV = 73.541; Scale est. = 51.506; n = 24				
**Number of shoots**~treatment + varieties + s (days, k = 6, by = treatment)				
Parametric coefficients:				
	Estimate	Std. Error	t value	Pr(>|t|)
(Intercept)	8.5208	0.7611	11.195	2.01 × 10^−9^ ***
**treatmentWW**	**6.2083**	**0.8789**	**7.064**	1.58 × 10^−6^ ***
**varieties Centauro**	**2.7083**	**0.8789**	**3.082**	0.00657 **
Approximate significance of smooth terms:				
	edf	Ref.df	F	*p*-value
**s (days): treatmentDS**	**1.126**	**1.240**	**36.16**	6.51 × 10^−6^ ***
**s (days): treatmentWW**	**2.307**	**2.828**	**88.20**	<2 × 10^−16^ ***
---				
Signif. codes: 0 ‘***’ 0.001 ‘**’ 0.01 ‘*’ 0.05 ‘.’ 0.1 ‘ ’ 1				
R-sq.(adj) = 0.938; Deviance explained = 95.3%				
GCV = 6.3317; Scale est. = 4.6344; n = 24				

**Table 4:** Forage quality: average values and relative standard deviation of quality parameters of cv. Bellante and cv. Centauro under drought stress (DS) and well-watered conditions (WW). Abbreviations: CT = condensed tannin, N = Total Nitrogen; DW = dry weight, CE, catechin equivalent, GAE = gallic acid equivalents, NDF = neutral detergent fiber.

	**Extractable CT (mg CE g^−1^ DW)**				**Protein-Bound CT (mg g^−1^ DW)**				**Fiber-Bound CT (mg g^−1^ DW)**				**Total CT (mg g^−1^ DW)**			
	**Bellante**		**Centauro**		**Bellante**		**Centauro**		**Bellante**		**Centauro**		**Bellante**		**Centauro**	
	**mean**	**sd**	**mean**	**sd**	**mean**	**sd**	**mean**	**sd**	**mean**	**sd**	**mean**	**sd**	**mean**	**sd**	**mean**	**sd**
DS	3.24 ± 0.16		4.37 ± 0.28		3.32 ± 1.55		3.15 ± 0.27		0.49 ± 0.26		0.52 ± 0.05		7.05 ± 1.17		8.04 ± 0.03	
WW	4.13 ± 0.10		2.68 ± 0.91		2.16 ± 0.57		2.49 ± 0.81		0.43 ± 0.14		0.38 ± 0.28		6.71 ± 0.60		5.55 ± 0.35	
	**Crude protein**				**NDF g 100 g^−1^ DW**				**Total Polyphenol (mg GAE g^−1^ DW)**							
	**Bellante**		**Centauro**		**Bellante**		**Centauro**		**Bellante**		**Centauro**					
	**mean**	**sd**	**mean**	**sd**	**mean**	**sd**	**mean**	**sd**	**mean**	**sd**	**mean**	**sd**				
DS	24.79 ± 3.95		24.93 ± 1.55		24.19 ± 2.44		25.23 ± 3.15		7.46 ± 1.2		6.67 ± 0.39					
WW	27.12 ± 1.52		29.75 ± 0.87		26.90 ± 1.25		25.83 ± 4.92		7.62 ± 2.93		6.31 ± 0.69					

**Table 5:** ANOVA of forage quality parameters. Parameters with *P* > 0.05 are non significant (ns).

Condensed Tannins	Drought Stress	Variety	Drought Stress × Variety
Extractable tannins	*P* = 0.05	ns	*P* = 0.000577
Protein bound tannins	ns	ns	ns
Fiber bound tannins	ns	ns	ns
Total tannins	*P* = 0.00304	ns	*P* = 0.01029
Total N	*P* = 0.0272	ns	ns
NDF	ns	ns	ns
Total Polyphenols	ns	ns	ns

## Data Availability

Data are available upon request to the corresponding author.
